# Stated Preferences of Doctors for Choosing a Job in Rural Areas of Peru: A Discrete Choice Experiment

**DOI:** 10.1371/journal.pone.0050567

**Published:** 2012-12-18

**Authors:** J. Jaime Miranda, Francisco Diez-Canseco, Claudia Lema, Andrés G. Lescano, Mylene Lagarde, Duane Blaauw, Luis Huicho

**Affiliations:** 1 CRONICAS Centre of Excellence in Chronic Diseases, Universidad Peruana Cayetano Heredia, Lima, Peru; 2 School of Medicine, Universidad Peruana Cayetano Heredia, Lima, Peru; 3 Salud Sin Límites Perú, Lima, Peru; 4 Department of Parasitology, and Public Health Training Program, US Naval Medical Research Unit 6 (NAMRU-6), Lima, Peru; 5 School of Public Health and Administration, Universidad Peruana Cayetano Heredia, Lima, Peru; 6 Department of Global Health and Development, Faculty of Public Health and Policy, London School of Hygiene and Tropical Medicine, London, United Kingdom; 7 Centre for Health Policy, School of Public Health, Faculty of Health Sciences, University of Witwatersrand, Johannesburg, South Africa; 8 School of Medicine, Universidad Nacional Mayor de San Marcos, Lima, Peru; 9 Instituto Nacional de Salud del Niño, Lima, Peru; World Health Organization, Switzerland

## Abstract

**Background:**

Doctors’ scarcity in rural areas remains a serious problem in Latin America and Peru. Few studies have explored job preferences of doctors working in underserved areas. We aimed to investigate doctors’ stated preferences for rural jobs.

**Methods and Findings:**

A labelled discrete choice experiment (DCE) was performed in Ayacucho, an underserved department of Peru. Preferences were assessed for three locations: rural community, Ayacucho city (Ayacucho’s capital) and other provincial capital city. Policy simulations were run to assess the effect of job attributes on uptake of a rural post. Multiple conditional logistic regressions were used to assess the relative importance of job attributes and of individual characteristics. A total of 102 doctors participated. They were five times more likely to choose a job post in Ayacucho city over a rural community (OR 4.97, 95%CI 1.2; 20.54). Salary increases and bonus points for specialization acted as incentives to choose a rural area, while increase in the number of years needed to get a permanent post acted as a disincentive. Being male and working in a hospital reduced considerably chances of choosing a rural job, while not living with a partner increased them. Policy simulations showed that a package of 75% salary increase, getting a permanent contract after two years in rural settings, and getting bonus points for further specialisation increased rural job uptake from 21% to 77%. A package of 50% salary increase plus bonus points for further specialisation would also increase the rural uptake from 21% to 52%.

**Conclusions:**

Doctors are five times more likely to favour a job in urban areas over rural settings. This strong preference needs to be overcome by future policies aimed at improving the scarcity of rural doctors. Some incentives, alone or combined, seem feasible and sustainable, whilst others may pose a high fiscal burden.

## Introduction

Human resources for health have risen as a public health concern in the international agenda [1], [2], and also as a national research priority in Peru [3], [4], where the health workforce is inequitably distributed [5], [6]. Density of doctors per 10,000 population is 7.7 in Lima, the capital city, and well below 4.0 and even close to 2.0 in most Andean and Amazon jungle departments, which together constitute the majority of rural areas of the country [5]. Likewise, analysing distribution by socioeconomic indicators, doctors are more concentrated in the richest area quintile (11.5 per 10,000 population) than in the poorest area quintile (1.9 per 10,000 population) [5], and large gaps exist in the distribution of specialised doctors across the country [7]. If an equitable distribution were to be achieved, 24% of doctors would have to be relocated [8].

Peru is therefore one of the few countries in the region facing a health workforce crisis, with health workers density below the critical threshold of 2.3 per 1,0000 population [9]. This crisis is not only evident in indicators of the geographical concentration of human resources [10], [11], but also in the perception of lay individuals. A recent survey of inhabitants of Lima, Peru’s capital, showed that they consider an insufficient number of doctors as the second most common problem affecting the quality of health service delivery [12]. Not surprisingly, if such a negative perception exists in the capital where doctors are mostly concentrated, the picture in remote and rural areas is likely to be worse. The task of attracting and retaining doctors to rural areas is particularly daunting and there is limited evidence on specific effective mechanisms to achieve this goal [1]. A combination of financial and non-financial incentives seems important, although the relative strength of particular incentives, alone or in combination, are most likely context-specific and different for different health cadres [1], [13], [14]. The evaluation of bundles of incentives has not been explored in Peru and could yield innovative results with direct policy implications.

In this study we report employment preferences of doctors for a post in under-served areas of Peru, obtained through a discrete choice experiment (DCE) [15], [16]. This approach allows the examination of preferences when individuals are forced to trade-off between certain job characteristics. It allows the assessment of the relative strength of the selected attributes, individually and in combination. It also facilitates running policy simulations that may be relevant for policymakers.

## Materials and Methods

### Ethic Statement

The study protocol and the informed consent forms were approved by the Ethics Committee of the Universidad Peruana Cayetano Heredia, Lima, Peru, by the Ethics Review Committee of the World Health Organization, Geneva, and by the Institutional Review Board of the US Naval Medical Research Unit No. 6 (NAMRU-6), Lima, Peru. Participation was voluntary and all participants signed the informed consent form before any study procedure.

### Setting

The Ayacucho department is divided into 11 provinces and each province into districts. Most inner and remote districts are rural areas, while the capitals of the provinces, including Huamanga, the department’s capital, constitute the urban areas of the department. Demographic and health surveys showed that the proportion of people living in rural areas was 64% in 2000, 60.8% in 2007–2008, and 58.9% in 2010 [17]–[19]. Thus recent figures indicate that most of the department’s population is still rural, although a dramatic rural-urban migration occurred in the 1980s and 1990s, boosted mainly by the political violence and social unrest that hit Peru and Ayacucho in particular during that period [20]. Our team selected seven provinces from the northern region of the Ayacucho department, where the poorest districts are concentrated [21]. Sociodemographic information on these provinces is shown in a related paper on nurses and midwives.

### Study Population

The public health sector is largely responsible for providing health care in Ayacucho department. Thus doctors working currently at the Ministry of Health facilities in this area were approached for this study. We focused on doctors on short-term contracts rather than on those with permanent posts because the latter group is comprised by more senior doctors, already settled in urban areas and therefore much less likely to consider moving to rural settings. Doctors identified were based in the only regional hospital of Ayacucho city, in health centers (usually in the periphery of Ayacucho city or in other provinces’ capitals such as Huanta and La Mar), and in health posts (mainly in rural and remote areas of the department). We included doctors working in urban or rural areas, because any attraction or retention strategy designed for doctors should forcefully target both groups. For those working in an urban area, attraction would be the initial goal, while retention would be the relevant policy issue for those already working in a rural area.

### Sampling

The department of Ayacucho as a whole has a low concentration of doctors [5], and a reliable updated list of professionals serving in the area is unavailable [22]. Thus our study team had to visit the rural and urban sites, and performed check procedures by contacting frontline informants in different health facilities, in order to assemble a preliminary list of all health personnel and assemble a sampling frame. A census of doctors working on short-term contracts, including those in their one-year rural service known as SERUMS [23], was conducted. This census was conducted in a total of 119 health facilities in seven provinces from the Ayacucho department, over a 7-week period, from September to October, 2010. Lists were further verified, to reconfirm counting of doctors who were travelling in the initial visit or changed their jobs. We attempted to obtain all doctors practicing in those facilities. A separate subgroup of doctors was approached at the regional hospital.

### Discrete Choice Experiment (DCE) Design

The identification of the most relevant attributes and levels to include in the DCE relied on several methods: in-depth interviews and focus groups performed with health providers currently working in Ayacucho [24], review of the international literature on attraction and retention strategies in low and middle-income countries [1], and interviews with policy makers.

A labelled discrete choice design was selected for this study and three labels of interest were identified – *rural community*, *other provincial capital city*, and *Ayacucho city* (Ayacucho’s capital). Job attributes were constructed according to these specific settings. A set of seven attributes were identified as potential determinant factors for doctors when choosing their preferred job package. These attributes were: type of health facility, monthly net income, time in post before getting a permanent job, points when applying for a residency in Community and Family Medicine after three years in post, provision of free housing, work schedule (excluding holidays), and number of allowance/free days for continuous medical education (Table 1). While our qualitative study performed before this DCE study found that nurses and midwives consistently expressed recognition for their jobs as a relevant incentive, this issue did not emerge strongly from the interviews and focus groups with doctors [24]. Therefore, we decided not to include this particular attribute in the final DCE design for doctors, whereas we included it in the final design for nurses and midwives.

**Table 1:** Final DCE design for doctors on short-term contract.

	RURAL COMMUNITY	AYACUCHO CITY	OTHER PROVINCIAL CAPITAL CITY
**1. Health facility**	• Health center	• Regional hospital	• General hospital or health center with inpatient beds
**2. Monthly take home (after tax) salary**	• S/. 2,500	• S/. 2,500	• S/. 2,500
	• S/. 3,125		• S/. 3,125
	• S/. 3,700		
	• S/. 4,375		
**3. Time in post before getting a** **permanent job**	• 3 years	• 6 years	• 4 years
	• 6 years	• 10 years	• 8 years
**4. Points when applying for a residency** **in Community and Family Medicine,** **after 3 years in post**	• 10 points bonus when applying fora residency in Community and Family Medicine	• None	• None
	• 20 points bonus when applying for a residency in Community and FamilyMedicine		• 10 points bonus when applying for a residency in Community and Family Medicine
**5. Free housing provided**	• A shared room in a residencewith shared facilities	• None	• None
	• A two-bedroom independent house		
**6. Work schedule (excluding holidays)**	• You work 22 days and thenhave 8 days off	• You work all daysexcept Sundays	• You work 22 days and then have 8 days off
	• You work 18 days and thenhave 12 days off		• You work 18 days and then have 12 days off
**7. Free days for continuous medical** **education**	• 7 free days a year	• None	• 7 free days a year
	• 14 free days a year		• 14 free days a year

The labelled design was chosen because it allows researchers to define different attributes and levels for the different labels, thus increasing the realism of the task and making it possible to define specific incentives for particular geographic areas [25], [26]. In addition, health workers had the option to choose neither of the job alternatives presented (‘opt out’). Final attributes and levels were determined through iterative discussions among the study team members and after piloting twice the DCE questionnaire prior to field application. The resulting final design, 16 choice-sets each with three different job options, is shown in Table 1.

Except for the salary attribute -which had four levels to allow for evaluation of nonlinear effects, all other attributes had 2 levels. This specification of attributes and levels resulted in a full factorial design with 8,192 combinations (i.e. 2 ^11^ x 4^1^). We used the macros developed by Kuhfeld [27] to select combinations for an orthogonal main effects design, and to organize the selected profiles into the most D-efficient choice design.

A questionnaire was developed to collect additional information on the socio-demographic and medical training characteristics as well as family, housing and labour circumstances of respondents that were thought to be influential on job choices. The DCE choice-sets and the socio-demographic questionnaire were applied to participants by a group of eight trained interviewers. Each participant was asked to place himself/herself in a hypothetical situation of looking for a new job, and in such context, different hypothetical job offers to work in the Ministry of Health’s facilities were presented to them.

### Statistical Analysis

We only analysed the responses of the forced choice questionnaire, as the proportion of participants who chose the opt-out option was small (7.4%). We used multiple conditional logistic regressions to assess the relative importance of job attributes and individual characteristics on job preferences. We calculated the odds ratios (OR) and their 95% confidence intervals (95%CI) for assessing the relative strength of job attributes and of participants’ individual characteristics. We further evaluated the preferences of different subgroups through the inclusion of interaction terms in the regression models. We then performed policy simulations, using our best-fitting estimation model, which allowed us to predict the impact of potential policy incentives on the probability of doctors choosing a rural job, a job in a small city, or a job in Ayacucho city. All analyses were conducted with Stata 11.0 for Windows (Stata Corp., College Station, TX).

## Results

### Sociodemographic Characteristics, Current Family and Work Circumstances

A total of 102 doctors, 63% males, participated in the study. Of these, 42 were based in Ayacucho city (41%), 14 in La Mar (14%), 14 in Huanta (14%) and 32 in other areas (31%). Overall, 59% of surveyed doctors considered their current post as a rural setting, 22% were born in the department of Ayacucho where they were currently working, only one had Quechua as his native language, none trained in medicine in Ayacucho, and on average they had 3.2 years of practicing medicine, 1.7 years of them in rural areas. Additional personal, family and work circumstances are presented in Table 2 and Table 3. While 50% reported that they did not want to continue in current post, only 42% would actually leave.

**Table 2:** Sociodemographic, medical training, family and housing characteristics of doctors on short-term contract (n = 102).

Sociodemographic			
Gender	Male	64	62.8
	Female	38	37.2
Native language	Spanish	101	99
	Quechua	1	1
Department of birth	Ayacucho	22	21.6
	Ica	35	34.3
	Lima	19	18.6
	Other	26	25.5
**Medical training and practicing experience**			
Department where trained	Ayacucho	0	0
	Ica	65	63.7
	Lima	15	14.7
	Other	22	21.6
Experience as doctor (years)*		3.2 (4.4)	
Experience as doctor in rural area (years)*		1.7 (2.1)	
**Current family and housing circumstances**			
Married/partner	Yes	40	39.2
	No	62	60.8
Lives with partner	Yes	16	15.7
	No	24	23.5
	No partner	62	60.8
Has children	Yes	39	38.2
	No	63	61.8
Housing type	Rents	66	64.7
	Owns	14	13.7
	Health facility	13	12.7
	Other	9	8.8

* Mean (±SD).

**Table 3:** Labour conditions of doctors on short-term contract (n = 102).

		N	%
**Workplace circumstances**			
Setting	Urban	42	41.2
	Rural	60	58.8
Type of facility	Hospital	17	16.7
	Health center	55	53.9
	Health post	30	29.4
Receives income from other sources (e.g. private practice)	Yes	38	37.2
	No	64	62.8
Contractual status	SERUMS (Contract)	39	38.2
	Contract	63	61.8
	Permanent staff	0	0
Life insurance provided	Yes	1	1
	No	99	97
	Do not know	2	2
Recognition for his/her work	Yes	43	42.2
	No	59	57.8
Believes he/she will continue in current post	Yes	13	12.8
	No	59	57.8
	Do not know	30	29.4
Wants to continue in current post	Yes	39	38.2
	No	51	50
	Do not know	12	11.8
**Participation in training activities**			
Currently training	Certificate program	49	48
	Masters	13	12.8
	Specialization	0	0
Ministry of Health trainings activities in 2010*		1.6 (2.2)	
Other training activities in 2010*		3.1 (3.2)	
**Salary and workload**			
Monthly workload by contract (hours)*		162 (16)	
Reported monthly workload (hours)		192 (32)	
Reported monthly workload (days)*		22.0 (3.0)	
Workload intensity (scale 0–10, 10 = max)*		6.7 (1.8)	
Monthly salary (Nuevos soles)*		2470 (545)	

* Mean (±SD).

### Job Preferences

Doctors were five times more likely to choose a job in the capital over one in a rural community (OR 4.97, 95% CI 1.2; 20.54), a strong evidence of preference for working in Ayacucho city (Table 4). However, there was no evidence of difference in the preferences for a job in a small city outside the capital, compared to a rural post. Each PEN S/. 1,000 nuevos soles increase in salary doubled the chances of choosing a job in a rural community and nearly tripled the chances of choosing a small city. Compared to this, bonus points for specialization appeared to be a positive albeit weak incentive for those in rural areas (OR 1.28, 95% CI 1.03; 1.57). There was evidence that the increase in the number of years needed to get a permanent contract acted as a significant disincentive in all three settings (Table 4). Job attributes that did not influence significantly on stated preferences for a rural job included type of free housing provided, monthly workload (days of work per month), and free days for continuous medical education training.

**Table 4:** Determinants of job preferences of doctors on a short-term contract.

	Odds Ratios	95%CI	p-value
**Alternative-specific constant**			
Ayacucho city	**4.97**	**1.20; 20.54**	**0.027**
Other provincial capital city	0.66	0.08; 5.25	0.698
**Rural job characteristics**			
Salary increase - per each S/. 1,000 nuevos soles	**2.07**	**1.77; 2.41**	**<0.001**
Years before getting a permanent job - per each year	**0.85**	**0.79; 0.91**	**<0.001**
Specialization - per each 10 points	**1.28**	**1.03; 1.57**	**0.023**
Independent house vs. shared room	1.02	0.82; 1.26	0.876
Days of work per month – per extra working day	0.99	0.94; 1.04	0.732
CME training - per additional annual free day	1.01	0.98; 1.04	0.546
**Urban job characteristics**			
Years before getting a permanent job - per each year	**0.91**	**0.85; 0.97**	**0.002**
**Ayacucho province job characteristics**			
Salary increase - per each S/. 1,000 nuevos soles	**2.82**	**1.97; 4.04**	**<0.001**
Years before getting a permanent job - per each year	**0.88**	**0.83; 0.93**	**<0.001**
Specialization - per each 10 points	1.11	0.88; 1.38	0.38
Days of work per month - per extra working day	0.96	0.91; 1.02	0.164
CME training - per additional annual free day	1.02	0.98; 1.05	0.333
	1.02	0.98; 1.05	0.333
**Interaction with rural label**			
Male	**0.66**	**0.52; 0.83**	**0.001**
Birthplace (Ica vs. Ayacucho)	1.05	0.79; 1.4	0.743
Birthplace (Lima vs. Ayacucho)	0.98	0.33; 2.91	0.964
Does not live with partner vs. does not have a partner	**1.47**	**1.11; 1.94**	**0.006**
Lives with partner vs. does not have a partner	0.8	0.58; 1.10	0.171
Years of experience, 2–4 vs. <2 yrs	1.14	0.80; 1.64	0.473
Years of experience, 5–7 vs. <2 yrs	0.81	0.51; 1.29	0.368
Years of experience, 8–14 vs. <2 yrs	0.92	0.57; 1.51	0.75
Paid serums vs. other (temporary or permanent)	1.05	0.71; 1.55	0.817
Salary within or above the offered range	0.96	0.72; 1.28	0.792
Hospital vs. health post/center	**0.27**	**0.19; 0.39**	**<0.001**
**N**	**102**		

Pseudo R^2^: 0.1262 Log-likelihood: −1567 Chi^2^ (25) = 452 p<0.001. CME: continuous medical education.

When interactions with the rural label were explored, only a few appeared to affect the preferences of choosing a job in a rural setting, namely sex, type of facility and living with a partner. Compared to females, being male reduced by 34% the chances of opting for a rural job. Similarly, the option of working in a hospital reduced those chances by 73%, compared to the alternative health post or health center. Conversely, those who reported not living with a partner were 47% more likely to prefer a rural post.

### Policy Simulations

Baseline characteristics were chosen to resemble real life job conditions in Peru at the time of the survey, and included the following: monthly salary PEN S/. 2,500 nuevos soles (∼US$ 900 dollars); six years in current post before getting a permanent job; no points when applying for a residency in Community and Family Medicine after three years in post; no free housing provided; contract with 26 working days; and no allowance/free days for continuous medical education.

**Figure 1:**
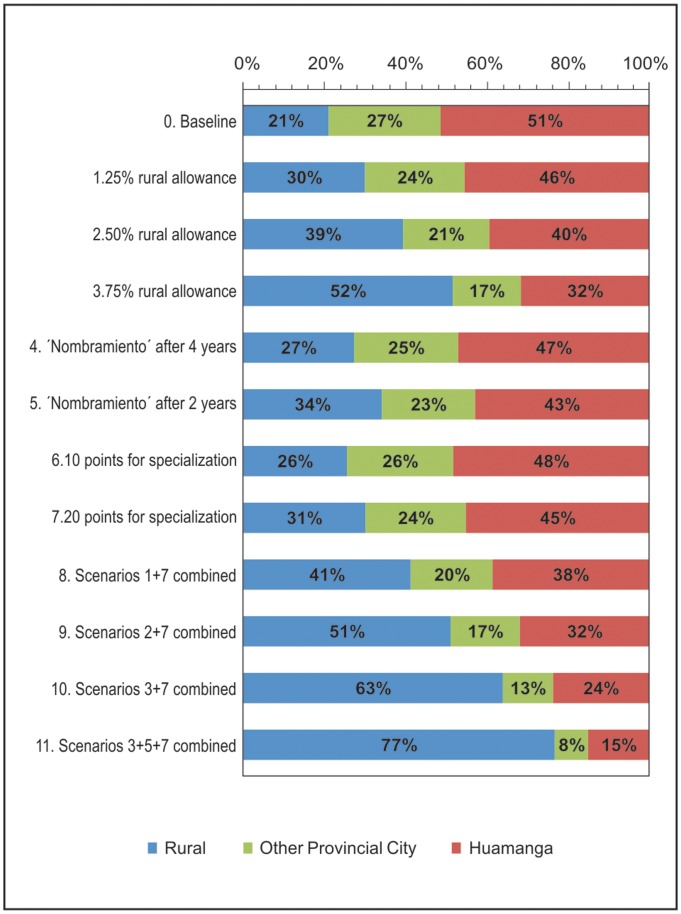
Policy simulations for doctors on a short-term contract showing changes in proportion opting for a rural job*. *These scenarios correspond to simulations when individual or combined incentives could be offered, relative to baseline scenario and using the coefficients of each specific attribute studied. The baseline scenario used for doctors included similar job conditions or characteristics for the three labels used (rural, small capital city, and Ayacucho city).

On the base scenario described above, 51% would opt for an urban job (Ayacucho city), 27% for a job in other provincial city and 21% would choose a rural post (Figure 1). The single most powerful intervention to affect this profile was an increase of salary. A 75% increase from baseline in salaries for those locating in rural settings would increase the uptake preference of a rural post to 52% (an increase of 31 percentage points from the baseline of 21%) while decreasing the urban uptake to 32%. Different alternatives, including combinations of incentives, were modelled to explore ways to reverse the urban predisposition. For example, increasing salaries by 50% and adding 20 bonus points when applying for a specialization course would yield the same proportion of doctors choosing a rural job as the scenario of 75% increase in salaries. The most dramatic simulation favouring the uptake of a rural post included a combination of 75% salary increase, getting a permanent contract after two years serving in rural settings and getting bonus points for further training, which resulted in an increase of rural uptake from 21% at baseline to 77% if these policies were to be introduced (an increase of 56 percentage points).

## Discussion

### Main Findings

Our study aimed to evaluate job preferences of doctors for working in under-served areas of Peru, assessing the relative strengths of several attributes and exploring policy simulations potentially relevant for policymakers. Our results indicate that doctors are five times more likely to choose an urban area than a rural one, despite the fact that both settings are located within the same department in the highlands of Ayacucho. This clearly shows the strength of doctors’ preferences for urban areas, and consequently the urgent need to develop initiatives to attract and to retain health workforce in rural under-served areas.

Although the context for reversing the situation is complex and difficult, our study shows that stand-alone incentives or combinations of them could improve the deployment of doctors in rural settings. These incentives would need to be considered by policy makers taking into account its feasibility, financial costs and long-term societal benefits for the population and for Peru’s rural development. For example, major increases in salary, in the order of 75% relative to their current ones, could act as an important incentive to reverse the current rural-urban imbalance in doctor workforce allocation. Combinations of scenarios and initiatives could also yield similar or even higher rates of attraction to rural areas and could still be politically feasible and sustainable.

### Comparison with Literature

The proportion of doctors working in rural areas in Peru is less than in urban settings [5], and a similar scenario occurs in neighbouring Brazil [28]. Our study was conducted with practicing doctors, 80% of them born in another department and all of them trained outside Ayacucho. Consequently, they already showed a life-story of relocation to Ayacucho, one of the most deprived departments of Peru.

In another study also conducted through a DCE, albeit with a sample of 39 doctors based in higher-level facilities and not necessarily in rural areas, Rockers et al. [29] explored preferences among Uganda’s health workforce to consider the uptake of a rural job. They found that doubling of salary, improvements to health facility quality, a contractual commitment to the posting for two years, and full tuition support for continued education at the end of this contractual commitment would make those postings more attractive [29]. Our findings are in the same direction, and have the advantage of exploring preferences of doctors currently placed in rural primary-level facilities and with short-term contracts.

In our case, instead of exploring a single best-case scenario, we modelled different strategies to better inform policy makers, taking into account the different nature, feasibility and scalability of potential strategies, including the variety of institutions required to implement them. We also collected information from those currently in service and with a trajectory of relocation to rural areas. Their input would be enormously valuable as their perceived benefits or incentives are likely to better inform policy makers. Such a policy exercise, previously reported for nurses in Kenya, South Africa and Thailand [25], is a better way of comparing the impact of different attributes in a DCE and is better for planning and decision-making processes, particularly in low-resource settings.

Other DCE exercises have been conducted in other low-income settings, for example with registered nurses in Malawi [30], where also a post in a city was more preferred over town, and salaries also appeared as the most valued attribute. Certainly incentives are complex and, to a great extent, context-specific [31]–[33]. In Vietnam [34], a qualitative research also found current salaries to act as a disincentive for workers currently placed in rural areas. Interestingly, one of the main motivating factors for health workers was appreciation by managers, colleagues and the community, a stable job, income, and training [34]. In our study, nearly 60% of respondents reported not having received the recognition they deserved for their work. Not surprisingly, a survey among recently graduated Peruvian doctors report that only one out of 14 considered working in inner parts of the country in the first five years following graduation and just one out of 200 considered doing so in rural areas [35].

### Relevance to Public Health

Findings from our research suggest a variety of possibilities to improve doctors’ deployment in rural settings. Peruvian health professionals’ wages decreased enormously, in particular during the economic crisis that Peru faced in recent decades, [36]–[38], and have not yet been completely reversed. Moreover, within the public health sector, higher salaries are observed for those who work for EsSalud, Peru’s social security system, compared with those who work for the Ministry of Health [39], and the latter is by far the main provider of the health workforce for rural areas. In urban areas, dual practice is a widespread activity that is well-accepted as a mean of financial income generation [40]. In our survey, 37% of participants reported having another source of income other than their medical salaries based on short-term contracts.

In this complex scenario, it is likely that financial incentives will need to be complemented with other types of incentives. In a fragmented health system such as the Peruvian one [41], this poses additional challenges. Chile for example, has been actively trying to improve attraction of doctors to rural health posts through its Rural Practitioner Programme that includes financial and non-financial incentives [42], and this experience offers lessons that could inform decision making processes in other similar settings including Peru.

So far, much of the research on human resources for health has been conducted in high-income settings [42], [43]. There have been a few more recent studies from low- and middle-income settings [44], [45]. Our study contributes towards closing this research gap. Ultimately, we expect that our findings provide new information related to some aspects contributing to this problem, in particular job preferences which influence the uptake of a rural post in Peru, and can contribute towards more evidence-based policies. In this regard, the definition of priorities for research needs to match resources [46], which would further inform planning and costing activities for human resources for health [47].

Importantly, isolated policies from isolated actors or institutions are not likely to fulfil the entire gap for more health workers in rural under-served areas. Part of the solutions should arise from revising and adapting the education of health professionals to meet local needs [48], [49], and more specifically medical education.

### Strengths and Limitations

Our study has some potential shortcomings. First, the stated preferences method used, even if it allowed us to determine the relative strengths of the different attributes explored, cannot fully anticipate the decisions eventually made by participants in real life situations. Only following them along time we will be able in a position to ascertain which job choices they actually made and in which specific circumstances. Second, the diverse intrinsic and extrinsic factors influencing job choices in real life situations are amenable to changes over time, and therefore the relative importance of the explored factors may vary, and this needs to be considered when designing attraction and retention policy interventions. Third, the department of Ayacucho as a whole has a low concentration of doctors, and virtually all deployed in the area are general practitioners [5]. For national mapping, when doctors are disaggregated by clinical specialties, the gap is greater in gynaecology and obstetrics, paediatrics, internal medicine and general surgery [7]. Our intention was not to study preferences according to clinical specialties since we aimed to fill the gap at the provision of primary care level. On a separate issue, a reliable updated list of professionals serving in the area was not available [22]. We overcame the latter obstacle by conducting a census of doctors currently working in the study region, that is very isolated areas, ranging from 2 to 7 hours drive from Ayacucho city, the capital. Finally, when informing policymakers about potentially useful incentives that could be included when planning attraction and retention strategies, it is critical to consider the financial and human resources burden that different combinations of incentives may exert on both the public budget as well as the Ministry of Health. Thus an ideal combination that includes all possible factors explored would require a substantial commitment from the government, and would be challenging under the currently prevailing circumstances.

### Conclusions

Doctors are five times more likely to favour a job in urban areas over rural settings. This observation sets a high bar to be overcome by future attraction and retention policies aimed at improving the scarcity of doctors in rural settings. Our study evaluated the magnitude of certain attributes that could counteract such a marked preference for urban posts. The number of days in service, time allocation for continuing medical education, and provision of free housing do not appear to be significant incentives in doctors’ preferences. Whilst salary increases above a certain threshold seem to represent an important incentive on its own, offering points for further specialisation and reducing the number of years before getting a permanent job in the sector could strengthen the positive effect of a salary increase.
